# Storage Optimization of Transmission Holographic Gratings in Photohydrogels

**DOI:** 10.1021/acsami.4c06436

**Published:** 2024-08-26

**Authors:** Kheloud Berramdane, María Isabel Lucío, Manuel G. Ramírez, Víctor Navarro-Fuster, María-José Bañuls, Ángel Maquieira, Marta Morales-Vidal, Augusto Beléndez, Inmaculada Pascual

**Affiliations:** † I. U. Física Aplicada a las Ciencias y las Tecnologías, Universidad de Alicante, Carretera San Vicente del Raspeig s/n, San Vicente del Raspeig 03690, Spain; ‡ Instituto Interuniversitario de Investigación de Reconocimiento Molecular y Desarrollo Tecnológico (IDM), Universitat Politècnica de València, Universitat de València, Camino de Vera s/n, Valencia 46022, Spain; § Departamento de Física, Ingeniería de Sistemas y Teoría de la Señal, Universidad de Alicante, San Vicente del Raspeig 03690, Spain; ∥ Departamento de Química, Universitat Politècnica de València, Camino de Vera s/n, Valencia 46022, Spain; ⊥ Departamento de Óptica, Farmacología y Anatomía, Universidad de Alicante, Carretera San Vicente del Raspeig s/n, San Vicente del Raspeig 03690, Spain

**Keywords:** photohydrogel, unslanted transmission volume phase holograms, diffraction efficiency, acrylamide-based hydrogel matrix, optimization

## Abstract

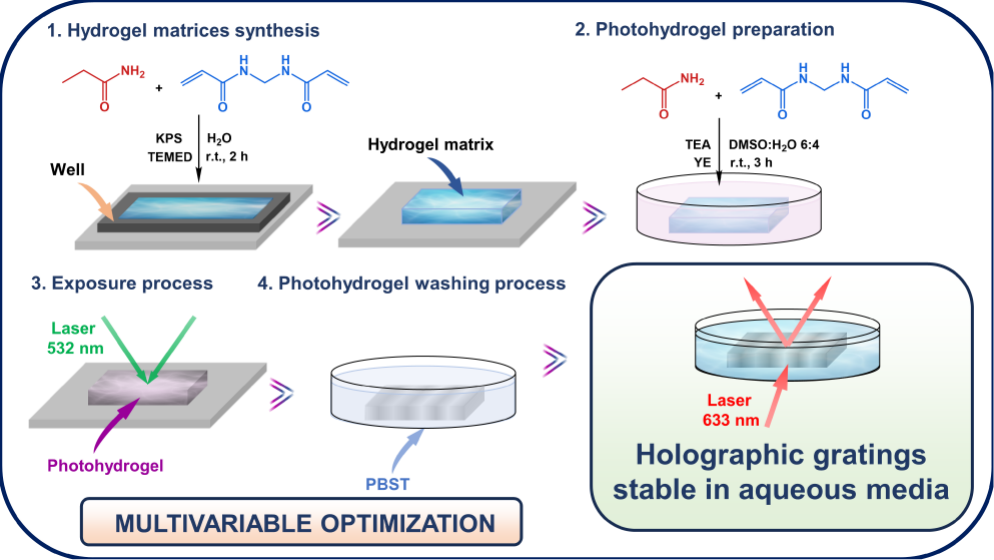

The development and optimization of holographic materials represent a great challenge today. These materials must be synthesized according to the characteristics that are desirable in photonic devices whose application is the object of investigation. In certain holographic sensors and biosensors, it is essential that the recording material be stable in liquid media. Furthermore, the holographic gratings stored in them must have temporal and structural stability, so that they can act as transducers of the analytical signal. Therefore, it is essential to optimize its storage in terms of the chemical composition of the material and the optical parameters of recording. This work focuses on the study of the storage optimization of unslanted transmission volume phase holograms in photohydrogels based on acrylamide and *N*,*N’*-methylenebis(acrylamide). Hydrogel matrices, also composed of acrylamide and *N*,*N’*-methylenebis(acrylamide), with different degrees of cross-linking were used and analyzed by scanning electron microscopy and UV–visible spectroscopy. The best results in terms of diffraction efficiency were reached for hydrogel matrices with an acrylamide/*N*,*N’*-methylenebis(acrylamide) molar ratio between 19.9 and 26. This relationship was also optimized in the incubator solution used to incorporate the components necessary for the formation of the holograms in the hydrogel matrices. The maximum diffraction efficiency, about 35%, was achieved when using an incubation solution with an acrylamide/*N*,*N’*-methylenebis(acrylamide) molar ratio of 4.35. The influence of the physical thickness of the hydrogel layers, the intensity, and the exposure time on the diffraction efficiency was also investigated and optimized. In addition, the behavior of the hologram was analyzed after a washing stage with PBST. A simple model that considered the effects of bending and attenuation of holographic gratings was proposed and used to obtain the optical parameters of the holograms.

## 1 Introduction

In the last decades, research in holography has acquired great relevance since it enables the manufacture of holographic devices and allows the storage of information through the volume of a photomaterial.^1^ Holography is an optical technique that allows for the storage and reconstruction of three-dimensional (3D) objects. The storage information in a recording material is obtained through the interference of two spatially overlapping coherent light beams. Holography finds multiple applications in sensing and biosensing,^2^ digital microscopy,^3^ data storage,^4^ 3D imaging,^5^ 3D displays,^6^ and optical tweezers,^7^ among others. These applications are valuable in many fields, such as agriculture, monitoring of environmental hazards, industry, military, entertainment imaging, and medical diagnostics. In recent years, both laser technologies and optical systems have reached a high level of sophistication. Therefore, it is necessary to concentrate efforts on developing and optimizing holographic materials. Desirable properties for holographic materials are the capability to create bright holograms; good light sensitivity; flat spatial frequency response; no absorption; no haze; no shrinkage; fast hologram formation; stability (environmental and light); and, when possible, industrial availability.^8^ Nonetheless, some of these ideal features are counterproductive for some specific eventual purposes. For example, no shrinkage is mandatory for data storage^9^ while shrinkage and/or swelling is desired to improve the sensitivity of holographic sensors.^10^ Different recording materials have been used for the hologram formation in past decades,^11^ including photopolymer, photorefractive glasses, silver halide emulsions, photoresists, and photopolymerizable glass.^12,13^ Photopolymers have very interesting properties such as high light sensitivity, relatively low-cost, self-processing capacity, and wide chemical versatility, and these properties render holograms of different composition with large dynamic ranges and high diffraction efficiency.^14^ Because of that, photopolymers are among the top preferred materials for the fabrication of optical elements^15−18^ or waveguides.^19,20^ Photopolymers require a polymeric matrix or binder for the formation of the hologram. Historically, poly(vinyl alcohol) (PVA) has been one of the most useful binders for photopolymers.^20,21^ However, the main handicap of the PVA-based materials is their sensitivity to humidity,^22^ which eventually results in the hologram destruction in aqueous environments.^23^ Therefore, PVA is not an adequate matrix for applications in watery solutions, such as the case of holographic sensing and biosensing in buffers. Holographic sensors are analytical devices that diffract light aimed to detect and quantify analytes or respond to physical stimuli.^2^ The interaction of the analyte with the hologram changes its refractive index modulation, its average refractive index, or/and its fringe space, which translates in an optical measurable analytical signal. One of their main advantages is that holographic sensors operate in a label-free format and can carry out direct detection in real-time. They have been applied in the detection of pH,^24,25^ humidity and temperature,^26^ metal ions,^27^ and glucose,^28,29^ and they have a huge potential for their use in point-of-care devices due, among other features, to their relatively low cost and wide spectral response. For this purpose, it is preferred to use hydrogel matrices as binders.^30^ Hydrogels are three-dimensional polymeric networks that retain a huge amount of water They can be chemically engineered to become sensitive so they can shrink or swell as a response to their environmental conditions.^31^ Thus, they have been used in controlled drug release systems,^32^ soft robotics^33^ or sensing.^34^ To date, researchers have deeply studied the storage of holograms with photopolymers,^35−37^ and we have recently shown how to process holographic hydrogels to obtain materials stable in liquid media.^38^ However, holographic gratings based on synthetic photohydrogels are yet a huge challenge currently, and systematic studies are still needed to understand all the parameters that influence the entire recording process. Generally, the diffraction efficiency (*DE*, the ratio between the irradiances of diffracted and incident beams) and the angular and wavelength selectivity are essential properties of holographic materials that should be controlled.^8^ For that, the thickness, the chemical composition of the photohydrogel, and the recording parameters should be managed.

The main objective of this work is to deeply study the storage of transmission holograms in photohydrogels. Acrylamide (AAm) and *N*,*N’*-methylenebis(acrylamide) (MBA) have been chosen as starting materials as they are among the most used monomers and cross-linkers for the fabrication of photonic hydrogels, which need high transparency and stability.^39^ Therefore, hydrogel matrices of different composition (cross-linking degree) and thicknesses will be prepared and characterized. The necessary components for the preparation of the photohydrogels are incorporated into the matrices. Then, recording of unslanted transmission volume phase holograms with different mixtures of these components is carried out. In this sense, the variation of the cross-linking degree will be studied both by varying the quantity of monomer (AAm) and by varying the quantity of cross-linker (MBA). During the recording, the radiant exposure, exposure time, and irradiance will be modified to evaluate their influence in the hologram formation. The optimization of the storage of the holograms will be evaluated by analysis of their diffraction efficiency and angular selectivity. Finally, washing of the most promising materials in aqueous buffer is carried out and their stability was analyzed. In order to understand the behavior of the holograms stored in the photohydrogels, a theoretical model was proposed to consider the effects of bending and attenuation of the holographic gratings in depth on the diffraction efficiency. This study will offer insight for the fabrication of holograms useful in liquid media, which could become perfect candidates for the design of holographic sensors and biosensors.

## 2 Experimental Section

**Scheme 1:**
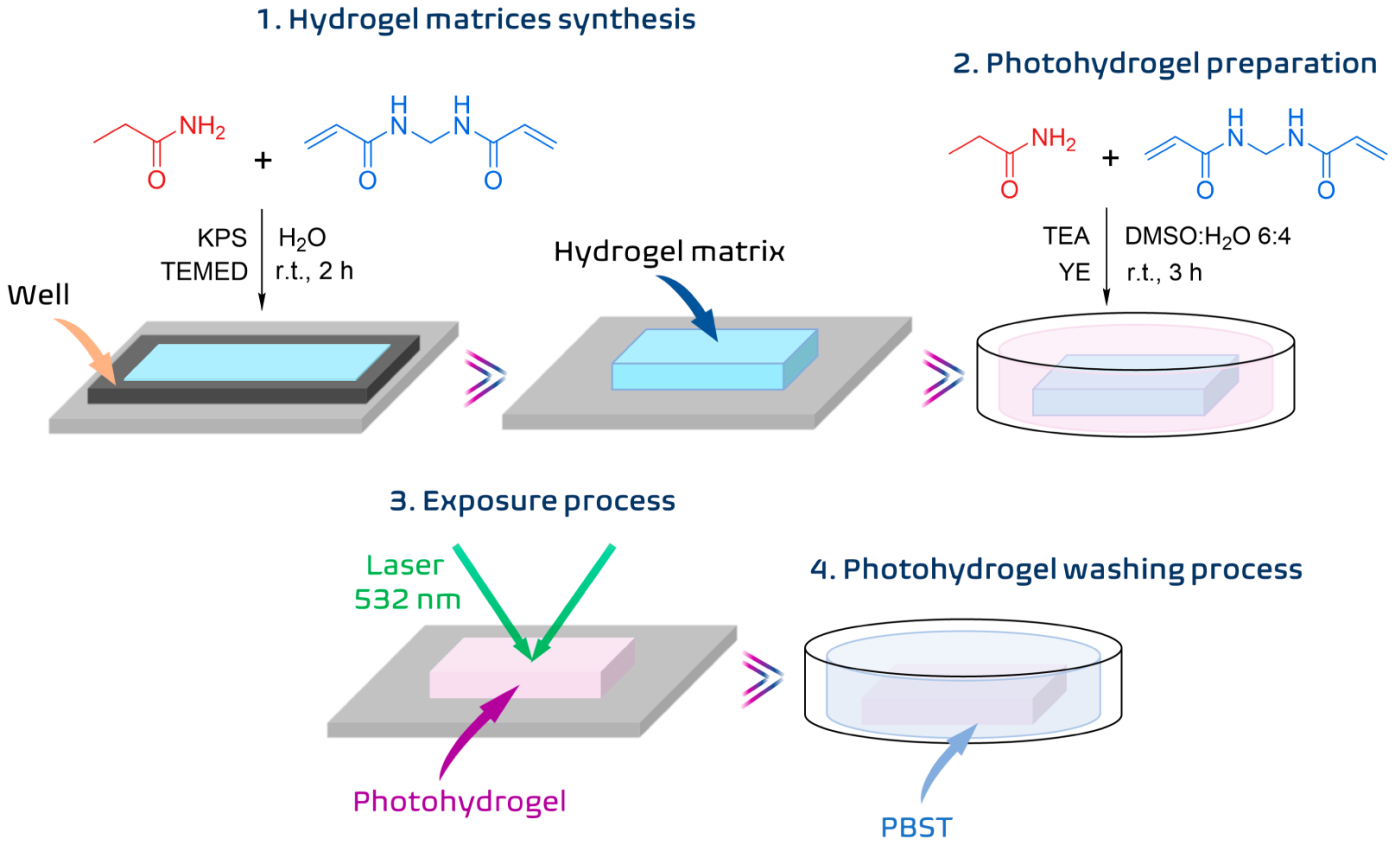
Procedures Carried Out for the Storage of Transmission Holographic Gratings in Photohydrogels KPS: potassium persulfate, TEMED: *N*,*N*,*N*′,*N*′-tetramethylethylenediamine, TEA: triethanolamine, YE: yellow eosin, DMSO: dimethyl sulfoxide, PBST: buffer.

The experimental process employed in this investigation is summarized in Scheme 1. Hydrogel matrices were synthesized at room temperature. Subsequently, these hydrogel matrices were immersed in a solution, and the necessary compounds to prepare the photohydrogels were incorporated. In the third part, unslanted transmission volume phase holographic gratings were stored in the photohydrogels through an exposure process with laser beams whose wavelength was 532 nm. Finally, a washing stage was carried out in order to remove the unreacted compounds in the exposure process and provide temporary stability to the stored holograms. The details of each process are described in the following sections.

### 2.1 Materials

Acrylamide (AAm), *N*,*N*′-methylenebis(acrylamide) (MBA), potassium persulfate (KPS), *N*,*N*,*N*′,*N*′-tetramethylethylenediamine (TEMED), triethanolamine (TEA), yellow eosin (YE), dimethyl sulfoxide (DMSO), potassium phosphate dibasic, potassium phosphate monobasic, sodium chloride, potassium chloride, and Tween-20 were purchased from Sigma-Aldrich Química S.L. (Madrid, Spain). PBST buffer 10 mM (pH 7.4) consists of potassium phosphate dibasic 0.8 mM, potassium phosphate monobasic 2 mM, sodium chloride 137 mM, potassium chloride 2.7 mM, and Tween-20 0.05% v/v.

### 2.2 Hydrogel Matrix Synthesis

Hydrogels matrices were prepared in thin layers using AAm as the monomer, MBA as the cross-linker, KPS as the initiator, and TEMED as the catalyst. AAm was dissolved in 1000 ± 2 μL of distilled water and different quantities of MBA were added to yield starting mixtures for obtaining hydrogels of different composition (Table 1). The parameter ω is defined as the AAm/MBA molar ratio. The solutions were homogenized by stirring during 1 h. KPS (10 mg) was then added, and they were sonicated for 2 min until the KPS was completely dissolved. The solutions were filtered through a 0.2 μm pore filter (Millipore, Burlington, Massachusetts, USA). Then, TEMED (2.40 ± 0.02 μL) was added, and the solution was homogeneously mixed by pipetting. 500 ± 1 μL of the resulted solutions were quickly deposited onto leveled glass slides (75 mm × 25 mm) (Labbox Labware, S.L., SLIBG10-050, Premia de Dalt, Spain) provided with sticking molds (Aironfix, Eon Paper S.L., Manresa, Spain) to create wells of 55 mm × 15 mm x 230 ± 20 μm. The systems were quickly sealed with another glass slide and tightened with two clamps. The hydrogels were allowed to polymerize at room temperature for 2 h. After the polymerization time, the hydrogels were peeled off, washed with distilled water, and stored in water at 4 °C. Hydrogels matrices F with ω = 22.6 ± 2.1 of different physical thicknesses were prepared using the same procedure but by depositing different volumes of the starting solution into wells of different thicknesses (500 ± 1 μL for 120 ± 20 μm, 500 ± 1 μL for 230 ± 10 μm, 500 ± 1 μL for 340 ± 20 μm, 1000 ± 2 μL for 460 ± 20 μm, and 1500 ± 6 μL for 570 ± 20 μm).

**Table 1:**
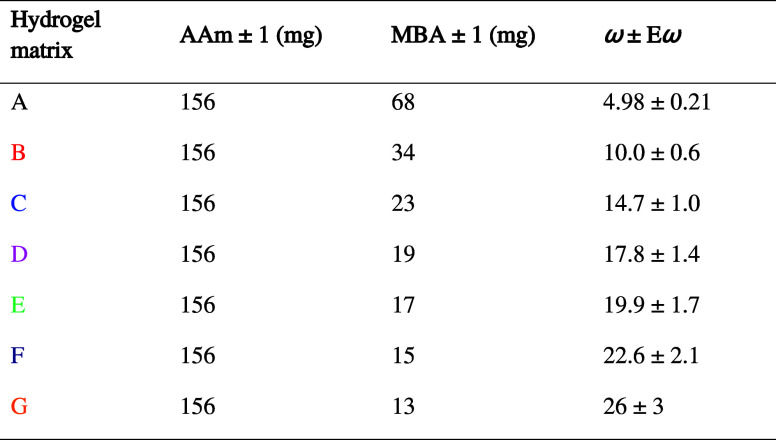
Composition of Hydrogels

### 2.3 Photohydrogel Preparation

Hydrogel layers (2.6 × 1.5 cm) were immersed for 3 h at room temperature in a Petri dish with 7.0 ± 0.1 mL of DMSO:H_2_O 6:4 (v/v) incubation solutions (IS) containing AAm, MBA, TEA, and YE. The amounts and concentrations in mole fraction (χ) of both AAm and MBA together with the parameter ω_IS_ (AAm/MBA molar ratio in the incubation solution) are summarized in Table 2. The volumes of TEA solution 0.995 ± 0.008 M, YE solution 8.0 ± 0.5 g/L, DMSO, and H_2_O were 1.00 ± 0.05, 0.220 ± 0.005, 6.00 ± 0.10, and 4.00 ± 0.05 mL for incubation solutions IS1 to IS5 and 1.75 ± 0.05, 0.390 ± 0.005, 10.50 ± 0.15, and 7.00 ± 0.10 mL for incubation solutions IS6 to IS9. After this time, the hydrogel layers (*n* ∼ 1.43 at λ = 589 nm) were placed onto flat glass slides (*n* = 1.4699 at λ = 632.8 nm, SLIB-G10-050, Labbox) for holographic exposure.

**Table 2:**
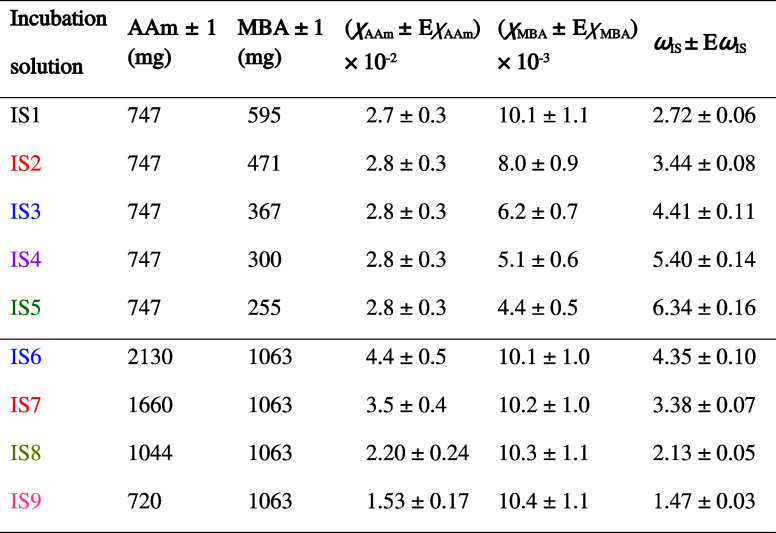
Compositions of the Incubation Solutions

### 2.4 Exposure Process

**Scheme 2:**
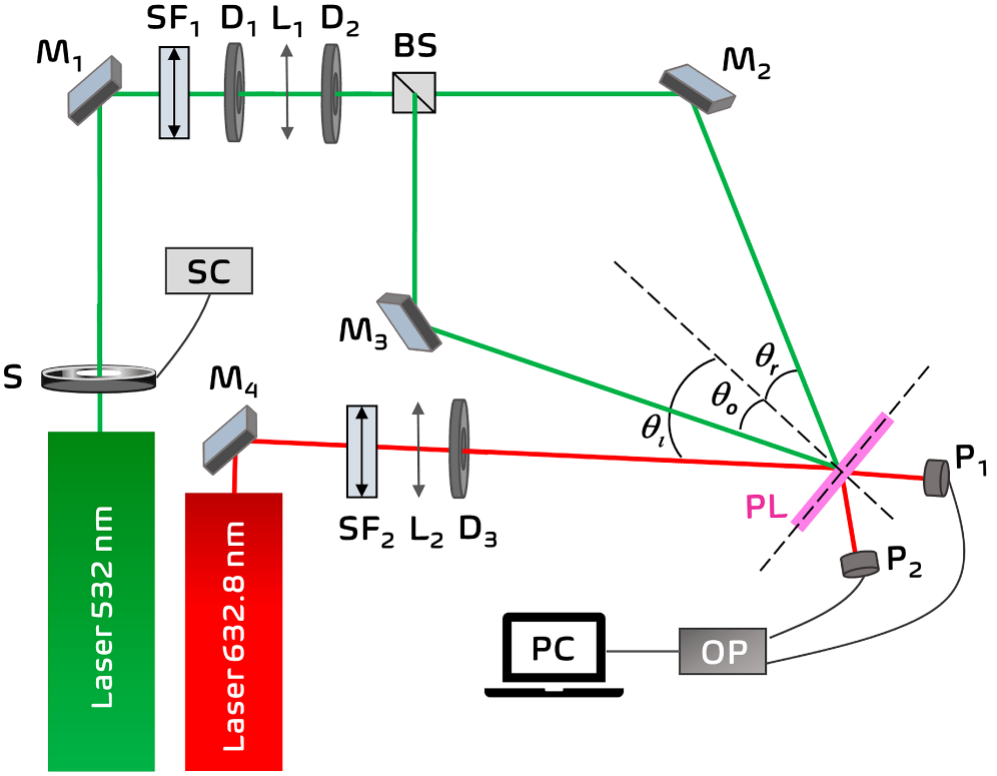
Holographic Setup for Transmission Gratings S: shutter; SC: shutter controller; BS: beam splitter; SF_i_: spatial filters (microscope objective and pinhole); Mi: mirrors; L_i_: lens; Di: diaphragms; θ_o_, θ_r_: object and reference recording angles; θ_i_: incident reconstruction angle; PL: photohydrogel layer; P_i_: photodetectors; OP: optical power meter; PC: data recorder.

The experimental holographic setup used for the exposure process of the unslanted transmission volume phase holographic gratings is shown in Scheme 2. The process was carried out under controlled light conditions, to which the photomaterial was not sensitive. A continuous (CW) Nd:YVO_4_ laser (Verdi-2W, Coherent, Santa Clara, CA, USA) emitting at λ = 532 nm was used. The laser beam was split into two secondary beams, object and reference beams, using a beam splitter (Newport, Irvine, CA, USA). The ratio of the intensities between both beams was 1:1. Then, the beams were spatially filtered and collimated. The diameters of both beams were 0.35 cm. The object and reference beams were spatially overlapped at the sample with the recording angles θ_o_ = θ_r_ = 18.7°, with respect to the normal incidence. The total irradiance (sum of the irradiance of both beams measured on the surface of the hydrogel film) was selected by varying the power of the Nd:YVO_4_ laser. The irradiance (*E*) of a beam was obtained by dividing its power by the area of the laser spot on the surface of the film. The laser beams had linear polarization perpendicular to the plane of incidence that allows optimal interference. The holograms were recorded at a theoretical spatial frequency of 1205 lines/mm (period Λ_th_ = 0.830 μm). A He–Ne laser (model 30995, REO, Boulder, CO, USA) positioned at 22.4° (theoretical Bragg angle for Λ_th_ = 0.830 μm) was used in the reconstruction stage to measure both diffracted and transmitted powers. This angle must be changed when the reconstruction after the washing stage is carried out due to the swelling effect of the photohydrogels. The He–Ne laser has linear polarization parallel to the plane of incidence.

### 2.5 Photohydrogel Washing Process

The photohydrogels with stored unslanted transmission holograms were repeatedly immersed for 5 min in 5 mL of PBST buffer to remove the unreacted compounds in the exposure stage. Six washing steps were necessary for the complete removal of this compounds.^38^ The angular scans were measured immediately after exposure and after the six washing steps. In order to preserve the holograms, the hydrogels were immersed in PBST and stored at 4 °C.

### 2.6 Scanning Electron Microscopy (SEM) and UV–visible Spectroscopy

The hydrogel layers in distilled water were frozen at −20 °C. Then, they were lyophilized overnight in a Telstar Lyoquest freeze-drier to yield dry aerogel samples. Dry samples were covered with a Au layer of about 15 nm in a BAL-TEC SCD 005 sputter coater (Leica Microsystems). Finally, dry hydrogel layers were analyzed by scanning electron microscopy with a Gemini SEM 500 system (Zeiss). The transmittance of hydrogel layers totally swollen in water was measured by UV–visible spectroscopy (V-650, Jasco, Spain).

## 3 Results and Discussion

### 3.1 Behavior of the Diffraction Efficiency as a Function of the Cross-Linking Degree of the Hydrogel Matrices

**Scheme 3:**
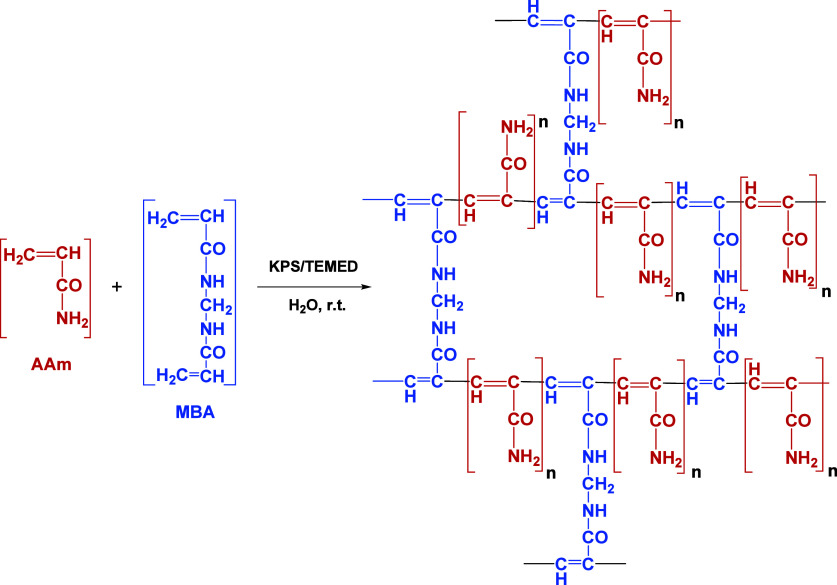
Chemical Structure of the AAm–MBA Matrix

**Figure 1:**
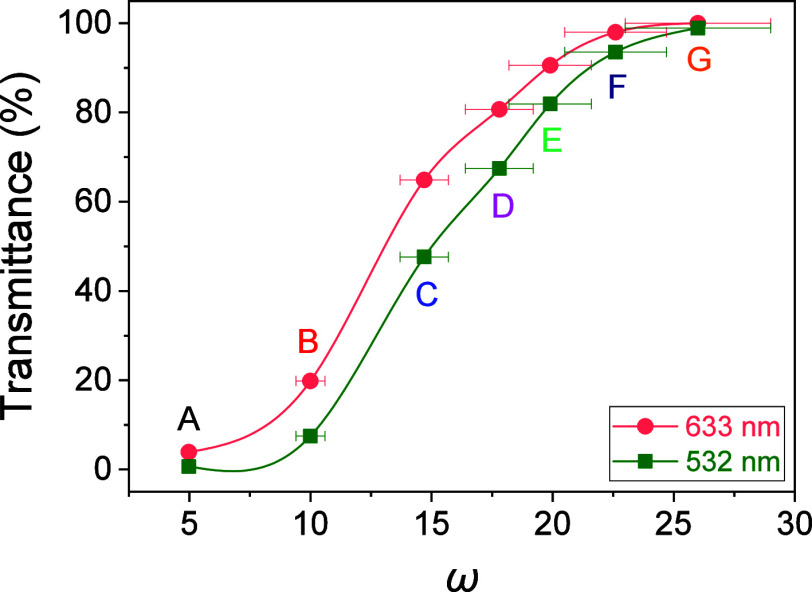
Transmittance of hydrogel matrices with different AAm/MBA molar ratios (ω). The well thickness was 230 ± 20 μm. Error is ±0.3% in transmittance. The solid lines are guides for the eye.

Hydrogel matrices based on AAm and MBA were synthesized as thin layers in order to store unslanted holograms in transmission and study their behavior, in terms of diffraction efficiency. The chemical structure of the AAm–MBA matrix is showed in Scheme 3. The characteristics of the matrix depend on the amounts of AAm and MBA used in its preparation, i.e., the AAm/MBA molar ratio (ω). The ω parameter can be related with the cross-linking degree. As the ω parameter decreases, the cross-linking degree increases. For hydrogels with the same quantity of AAm, the cross-linking degree will increase as the MBA increases. The cross-linking degree is a key parameter in hydrogels as it can affect multiple parameters such as their swelling or mechanical and optical properties. For the recording of holograms, ω is especially important as it will affect to the diffusion of the components^40^ that will later form the holographic gratings.^41^ For this study, hydrogel matrices of different compositions (Table 1) were synthesized and completely characterized. Figure 1 shows the transmittance obtained from the UV–visible spectra of hydrogel layers totally swollen in water at 532 and 633 nm as a function of ω. For comparison, all hydrogel matrices were prepared in wells with a thickness of 230 ± 20 μm. The wavelength values were chosen because they are the ones that will be used for holographic exposure and reconstruction, respectively. As it can be observed, the transmittance of the materials increases as the ω increases, i.e., the smaller the cross-linking degree, the higher their transparency.

**Figure 2:**
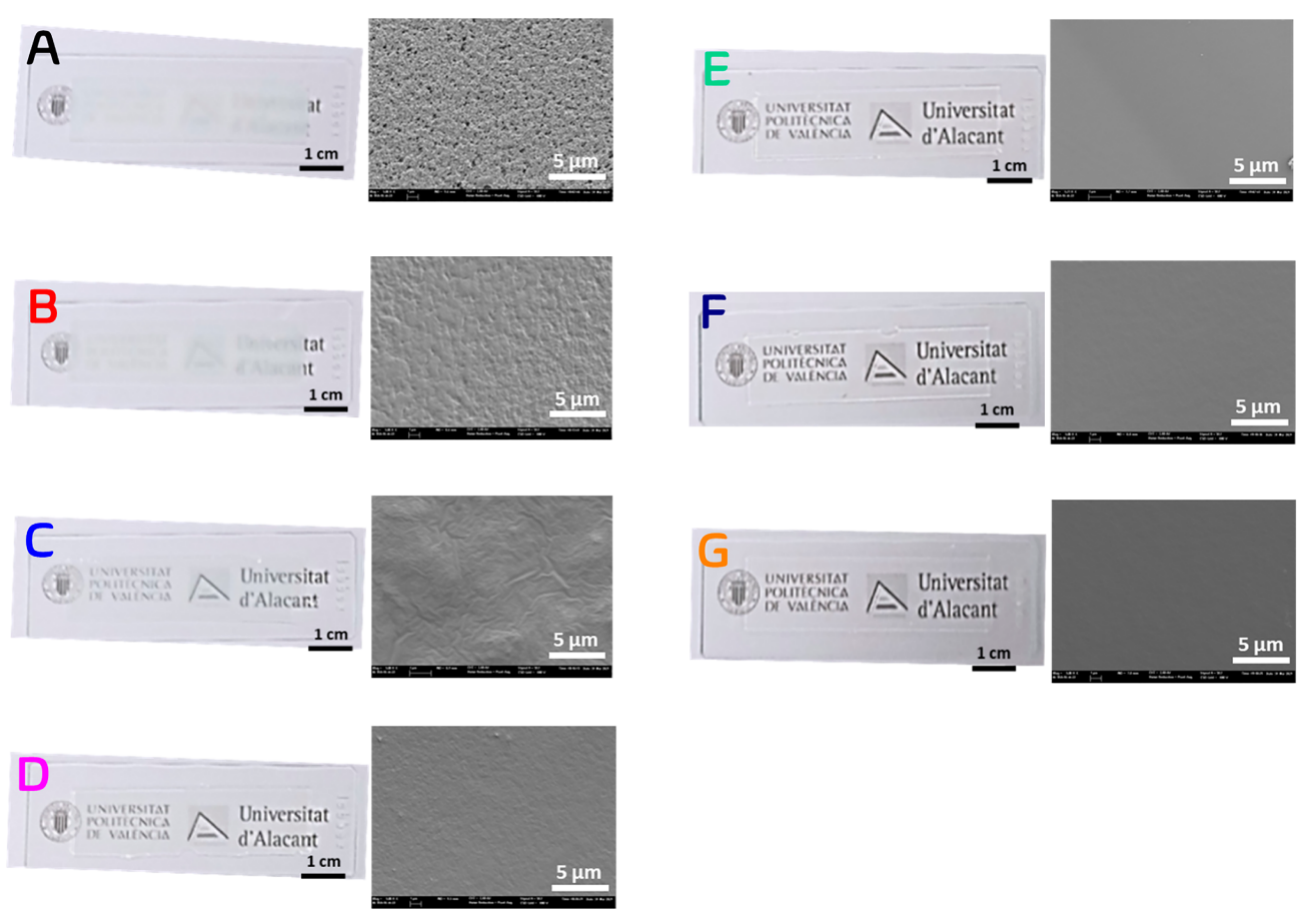
Film and SEM images of hydrogel matrices prepared in wells with a thickness of 230 ± 20 μm. Composition of the hydrogel matrices (A–G) is listed in Table 1.

Figure 2 includes digital images of the fully hydrated hydrogel layers. The same trend in transparency can be directly observed by the naked eye with the logos of our universities almost entirely hidden by the hydrogels with the highest cross-linking degree (hydrogels A and B). The highest transparency was obtained for hydrogels F and G. The water content in the layers was calculated as their swelling degree, , where *W*_0_ is the weight of freeze-dried hydrogel layers and *W*_t_ is the weight of the layers after their immersion in water for 48 h. *SD* values were 470% ± 30%, 469% ± 35%, 483% ± 12%, 521% ± 16%, 502% ± 11%, 489% ± 15%, and 532% ± 28% for hydrogels A–G, respectively. The internal morphology of the hydrogel films was analyzed by scanning electron microscopy. For that, completely swollen hydrogel films in water were freeze-dried to preserve their structural characteristics. Figure 2 also shows the SEM images of the dry hydrogels (0% water content). All hydrogels reveal a homogeneous nonmicroporous structure. However, pores of nanometric size can be observed in the hydrogels of the highest cross-linking degrees (hydrogels A and B). These heterogeneities can cause light scattering,^42^ which agrees with the opacity observed in the spectroscopic analysis.

**Figure 3:**
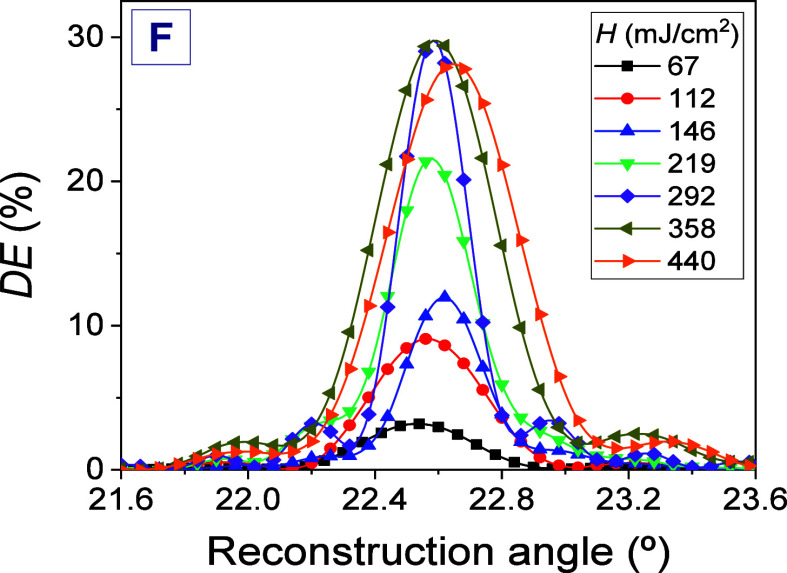
*DE* as a function of the reconstruction angle for hydrogel matrices F exposed with radiant exposure of 67 ± 3 (black box solids), 112 ± 4 (red circle solids), 146 ± 5 (blue triangle up solids), 219 ± 8 (green triangle down solids), 292 ± 10 (violet tilted square solids), 358 ± 11 (dark yellow triangle left-pointing solids), and 440 ± 10 (orange triangle right-pointing solids). The total irradiance was fixed to 7.29 ± 0.23 mW/cm^2^ and the exposure times were changed. ω_IS_ was 2.72 ± 0.06. The well thickness was 230 ± 20 μm. The solid lines are guides to the eye.

Photohydrogels were prepared following the procedure described in section 2.3. All hydrogel matrices were immersed in IS1 for 3 h. Subsequently, transmission holograms were stored in the photohydrogels. The total *E* was fixed to 7.29 ± 0.23 mW/cm^2^, and the exposure times (*t*_exp_) were changed to obtain different values of radiant exposure (*H* = *E*·*t*_exp_). In all cases, ω_IS_ was 2.72 ± 0.06. In this way, the cross-linking in the hydrogel matrix is the only parameter that was changed. Angular responses for each value of *H* were obtained. As an example, Figure 3 shows these angular responses when the hydrogel matrix F was used for the preparation of the photohydrogel. As can be seen, the Bragg angle at which the maximum *DE* is obtained presents small variations depending on the *H* used. This behavior is generalized for all types of hydrogel matrices used in this research (data not shown). For the F-IS1 photohydrogel, the Bragg angle varies from a minimum of 22.5° when *H* = 67 ± 3 mJ/cm^2^ was used up to a maximum of 22.6° for a *H* of 440 ± 10 mJ/cm^2^. A possible relationship between the Bragg angle variation and the *H* values for the different types of hydrogel matrices used in this work has not been observed. For this reason and in order to carry out a correct optimization study, we decided to measure the *DE* values in the Bragg angle (*DE*_B_) as a function of *H* through the angular responses for sets of photohydrogels based on the same matrices subjected to the same incubation conditions. This way, although longer, allows obtaining a statistically more reliable response compared to that in which the curves *DE* as a function of *H* are measured in real time for a single sample of photohydrogel.

**Figure 4:**
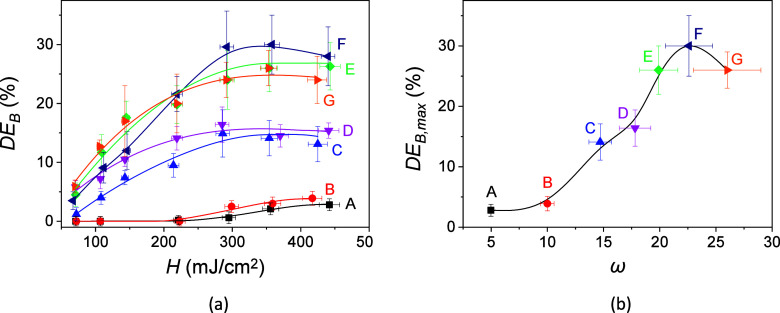
(a) *DE*_B_ as a function of radiant exposure for the different hydrogel matrices synthesized. ω were 4.98 (A, black box solids), 10.0 (B, red circle solids), 14.7 (C, blue triangle up solids), 17.8 (D, pink triangle down solids), 19.9 (E, green tilted square solids), 22.6 (F, navy triangle left-pointing solids), and 26 (G, orange triangle right-pointing solids). (b) *DE*_B,max_ achieved as a function of ω in the hydrogel matrix. The total irradiance was fixed to 7.29 ± 0.23 mW/cm^2^ and the exposure times are changed. ω_IS_ was 2.72 ± 0.06. The well thickness was 230 ± 20 μm. The solid lines are guides to the eye.

Figure 4a shows the *DE*_B_ values as a function of H for holograms stored in photohydrogels prepared from the seven types of hydrogel matrices investigated. As can be clearly observed, different behaviors are obtained depending on the type of matrix. For hydrogel matrices A and B, an exposure of about 221 ± 7 mJ/cm^2^ is required to begin to obtain a diffraction response. This means that there is an inhibition time of 30.3 s (the time in which the photomaterial does not present diffraction efficiency). The inhibition effects have been previously studied on acrylamide-based photopolymeric materials.^43^ This behavior is explained based on inhibitory agents, such as dissolved oxygen inside the photomaterials, that suppress the formation of radicals. The inhibition effects can be overcome by preserving the photomaterial from the atmosphere and increasing the exposure intensities. In our case, since the hydrogel matrices are initially stored in aqueous medium and later immersed in incubating solutions, the concentration of dissolved oxygen in the liquid medium is a parameter that must be studied and controlled. This phenomenon will be considered in future research. In addition, matrices A and B present a lower transmittance for the wavelength used in the recording stage, 532 nm (Figure 1). This causes the laser beams to have a greater attenuation when passing through the photohydrogel, and therefore, a greater radiant exposure is required to obtain diffraction. On the other hand, and as previously commented from the SEM images (Figure 2), matrices A and B present a porous structure that affects the diffusion processes of the components of the incubation solution inside of the hydrogel matrix and cause light scattering.^42^ These phenomena lead to a lower response, in terms of *DE*. Regarding the energy sensitivity, i.e., minimum energy per surface unit necessary to reach the maximum *DE*, values of 442 ± 15 and 417 ± 14 mJ/cm^2^ are obtained for matrices A and B while it is around 358 ± 12 mJ/cm^2^ for the rest of the matrices.

The slope in the initial sections of the *DE*_B_ curves as a function of *H* shows the rates of the photopolymerization processes. From Figure 4a, a clear difference can be observed depending on the degree of cross-linking of the matrix used for the preparation of the photohydrogel. In the interval of *H* between 221 and 356 mJ/cm^2^, slopes of 0.025 and 0.032 are obtained for matrices A and B, respectively. For matrices C and D, the slopes increase to values between 0.070 and 0.087 in the interval of *H* between 67 and 146 mJ/cm^2^. For this same interval of *H*, similar slopes of around 0.152 are measured for matrices E, F, and G. These results can be justified taking into account that the diffusion of the components of the incubation solution inside the hydrogel is favored as the degree of cross-linking of the matrix decreases. The maximum *DE*_B_ (*DE*_B,max_) measured are represented in Figure 4b as a function of ω for each type of hydrogel matrix. *DE*_B_ values of 2.8 ± 1.0 and 3.9% ± 1.2% were measured for matrices A and B, respectively. From these values, an increase is observed for matrices C and D for which similar *DE*_B,max_ of 14% ± 3% and 16% ± 3% were obtained, respectively. The highest *DE*_B,max_ was measured for matrices E, F, and G, with the lower cross-linking degree, reaching a maximum for matrix F for which a *DE*_B,max_ of 30% ± 5% was achieved. Taking into account the statistical errors calculated for the *DE*_B,max_ values, matrices E, F, and G were selected to continue the study.

### 3.2 Behavior of the Diffraction Efficiency as a Function of AAm and MBA Concentration in the Incubation Solution

Previous studies on AAm-based photomaterials have shown an enhancement of *DE* when MBA is added as a cross-linker.^44−46^ The use of MBA in hydrophilic acrylamide photopolymers enables the energetic sensitivity of the photomaterial compared to that achieved in photopolymers without cross-linkers. However, from a holographic point of view, a detailed study to optimize the AAm/MBA molar ratio in photomaterials based on hydrogels has not been carried out. The best hydrogel matrices in terms of *DE* selected in the previous section, E, F and G, were used to optimize the concentrations of AAm and MBA in the incubation solution. For this, and in order to cover a wide range of concentrations of both compounds, this stage of the investigation was divided into two parts. First, the number of MBA moles was varied, and the AAm moles remained fixed. Second, the amount of AAm moles varied, and the MBA moles were kept constant.

#### 3.2.1 Variation of the Amount of MBA in the Incubation Solutions

**Figure 5:**
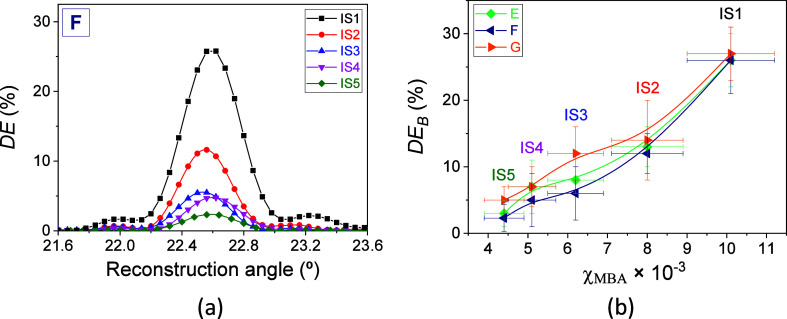
(a) *DE* as a function of the reconstruction angle for transmission gratings stored in photohydrogels prepared from hydrogel matrices F incubated in solutions with χ_MBA_ of 10.1 × 10^–3^ (IS1, black box solids), 8.0 × 10^–3^ (IS2, red circle solids), 6.2 × 10^–3^ (IS3, blue triangle up solids), 5.1 × 10^–3^ (IS4, pink triangle down solids), and 4.4 × 10^–3^ (IS5, green tilted square solids). χ_AAm_ was kept fixed at 2.8 × 10^–2^. (b) *DE*_B_ as a function of χ_MBA_ in the incubation solution for hydrogel matrices E (green tilted square solids), F (navy triangle left-pointing solids), and G (orange triangle right-pointing solids). The exposure time and total irradiance were 50.0 ± 0.1 s and 7.29 ± 0.23 mW/cm^2^, respectively (*H* = 365 ± 12 mJ/cm^2^). The well thickness was 230 ± 20 μm. The solid lines are guides to the eye.

Five incubation solutions, IS1-IS5, were prepared (Table 2). Due to the limited solubility of MBA in water, incubation solutions with χ_MBA_ higher than that used in IS1 were not used. In this way, MBA crystallization problems inside the photohydrogel were avoided. Hydrogel matrices E, F, and G synthesized in wells with a thickness of 230 ± 20 μm were immersed for 3 h in these ISs. The exposure time and total irradiance used were 50.0 ± 0.1 s and 7.29 ± 0.23 mW/cm^2^, respectively, which translate into an *H* of 365 ± 12 mJ/cm^2^. This value was selected in order to compare all photohydrogels with a radiant exposure slightly higher than their energy sensitivity, ensuring that in all cases the maximum *DE*_B_ is achieved. The angular responses of all stored transmission holograms were measured as a function of the MBA mole fraction in the IS. Figure 5a shows the angular responses when matrix F is used in the preparation of a series of photohydrogels. As can be seen, the *DE* grows as the χ_MBA_ increases. Noise gratings due to light diffusion phenomena were not observed. Since the moles of AAm remain fixed, the decrease in the amount of MBA leads to less cross-linking of the acrylamide molecules during the exposure stage in those areas where the interference of the laser beams is constructive. This translates into a lower modulation of the refractive index and consequently a decrease in diffraction efficiency. The *DE*_B_ was obtained from the angular responses and represented as a function of the composition of the IS for hydrogel matrices E, F, and G. The results are shown in Figure 5b. As observed, the behavior of *DE*_B_ was similar for the three types of matrices. As the concentration of MBA increases in the incubation solution, compositions IS2–IS5, the *DE*_B_ increases in an approximately exponential trend. The maximum values of *DE*_B_ are obtained when IS1, with the highest concentration of MBA, was used. These values correspond to 26% ± 4%, 26% ± 5%, and 27% ± 4% for the hydrogel matrices E, F, and G, respectively.

#### 3.2.2 Variation of the Amount of AAm in the Incubation Solutions

**Figure 6:**
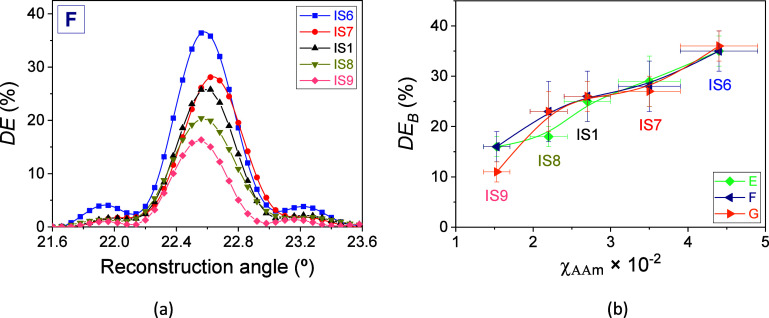
(a) *DE* as a function of the reconstruction angle for transmission gratings stored in photohydrogels prepared from hydrogel matrices F incubated in solutions with χ_AAm_ of 4.4 × 10^–2^ (IS6, blue box solids), 3.5 × 10^–2^ (IS7, red circle solids), 2.7 × 10^–2^ (IS1, black triangle up solids), 2.20 × 10^–2^ (IS8, dark yellow triangle down solids), and 1.53 × 10^–2^ (IS9, clear red square solids). χ_MBA_ was kept fixed at a value between 10.1 × 10^–3^ and 10.4 × 10^–3^. (b) *DE*_B_ as a function of χ_AAm_ in the incubation solution for hydrogel matrices E (green rhombus), F (navy triangle left), and G (orange triangle right). The exposure time and total irradiance were 50.0 ± 0.1 s and 7.29 ± 0.23 mW/cm^2^, respectively (*H* = 365 ± 12 mJ/cm^2^). The well thickness was 230 ± 20 μm. The solid lines are guides to the eye.

The composition of IS1 was taken as the starting point for the study of the variation of AAm in the incubation solutions. The moles of MBA were kept constant, while the amount of AAm was varied. Photohydrogels were prepared by immersing matrices E, F, and G for 3 h in incubation solutions IS6–IS9 (Table 2). ISs with higher amounts of AAm than that employed in IS6 were not considered as precipitation of AAm occurred on the surface of the hydrogel matrix. The conditions in the exposure stage were the same as those used in the preceding section. The angular responses of the transmission holograms stored in a series of photohydrogels are shown in Figure 6a when an F matrix was used. As can clearly be observed, *DE* grows as the χ_AAm_ increases from IS9 to IS6. The values of *DE*_B_ as a function of χ_AAm_ are represented in Figure 6b. A linear trend of the *DE*_B_ values is observed for the three types of matrices used in the photohydrogels. Maximum *DE*_B_ values of 35% ± 3%, 35% ± 4%, and 36% ± 4% are reached for matrices E, F, and G, respectively, when IS6 was used. It is important to note that the values of ω_IS_ are similar for the compositions of IS3 and IS6 as well as for IS2 and IS7. However, from Figures 5b and 6b, a notable difference in the *DE*_B_ values is observed. This behavior is due to the dilution of the AAm and MBA molecules inside the hydrogel matrices. The concentrations of these components in compositions IS6 and IS7 are higher than those prepared in IS3 and IS2. The value of ω_IS_ for the IS6 composition is 4.35 ± 0.10. Optimized values of the AAm/MBA molar ratio between 7.75 and 15.5 can be found in poly(vinyl alcohol) photopolymers based on AAm and MBA with the same photoinitiator system depending on the thickness used.^44,46^ In view of the results obtained for our photohydrogels, an increase in the ω_IS_ value accompanied by an increase in the concentration of AAm and MBA without precipitation and crystallization effects of both components could enhance the *DE*_B_ obtained, which motivates further future studies.

### 3.3 Dependence of Hydrogel Matrix Physical Thickness on the Diffraction Efficiency and Angular Bandwidth

**Figure 7:**
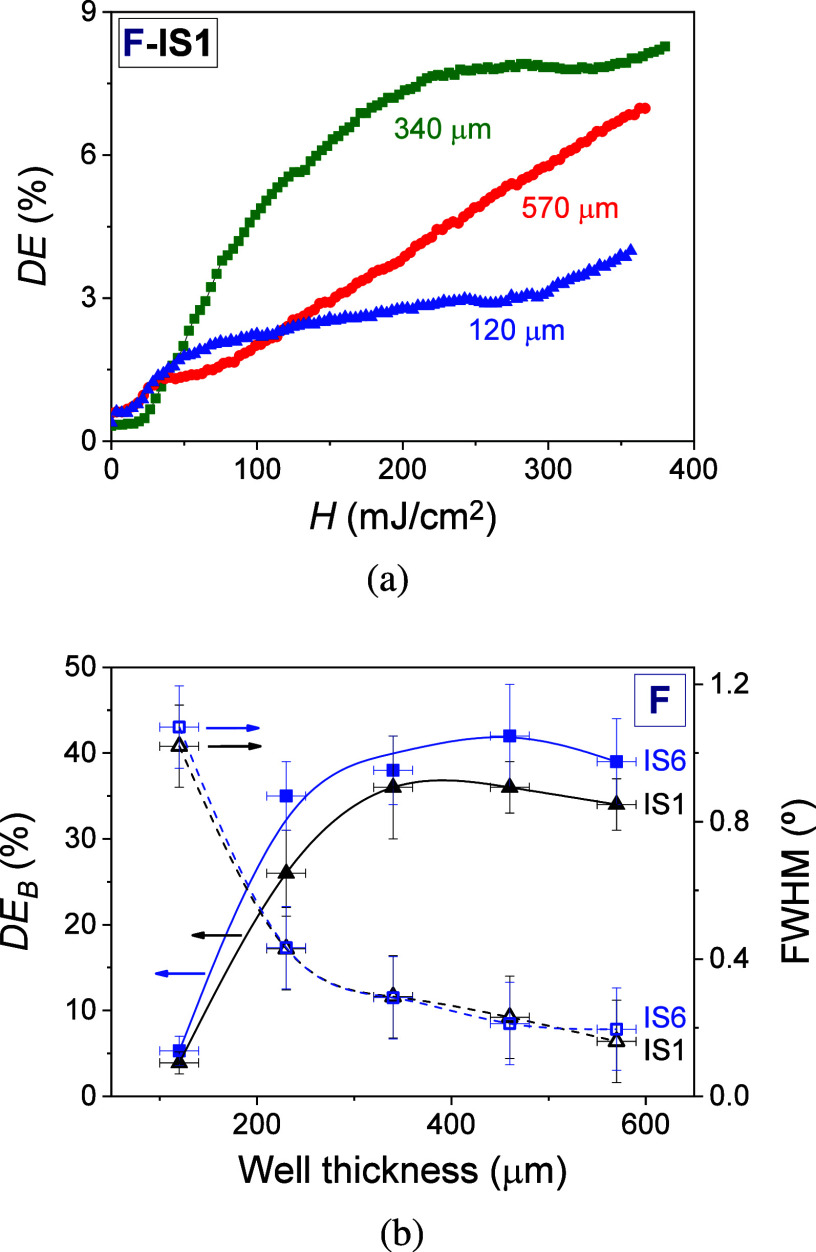
(a) *DE* as a function of radiant exposure for photohydrogels with well thicknesses of 120 (blue triangle up solids), 340 (green box solids), and 570 μm (red circle solids) when IS1 was used for the incubation stage. (b) *DE*_B_ (left axis) and FWHM (right axis) as a function of the mold physical thickness when IS1 (black triangles and lines) and IS6 (blue squares and lines) were used for the incubation stage. The transmission gratings stored in photohydrogels were prepared from hydrogel matrices F. The exposure time and total irradiance were 50.0 ± 0.1 s and 7.29 ± 0.23 mW/cm^2^, respectively (*H* = 365 ± 12 mJ/cm^2^). The solid and dashed lines are guides to the eye.

The dependence of the matrix physical thickness on the energy sensitivity in photomaterials based on AAm and MBA with poly(vinyl alcohol) polymer matrices has been previously studied.^46^ In these photomaterials, an increase in the energy sensitivity with the thickness of the layer was observed when their compositions were optimized. In this context, the influence of the physical thickness of our hydrogel matrices on the *DE* was investigated using the well thickness as the study parameter. It is more reliable to measure the well thickness than the physical thickness of hydrogel layers due to their physical characteristics. Figure 7a shows the diffraction efficiency as a function of radiant exposure measured in real time during the exposure stage for three F-IS1 photohydrogels prepared from wells with thicknesses of 120, 340, and 570 ± 20 μm. In order to observe the behavior of *DE*, only IS1 was used for the incubation. Note that the 633 nm beam is positioned at 22.4° during the exposure stage. Due to the angular variations between this angle and the Bragg angles obtained for each photohydrogel (as indicated in the previous sections 3.13.1and 3.23.2), discrepancies between the *DE* measured in real time and the *DE*_B_ were obtained. Despite this, the behavior of the holograms can be followed in Figure 7a. Different behavior is observed for the three well thicknesses and a clear difference in their energetic sensitivities cannot be described. For this reason, the same conditions for the exposure stage as those used in the previous section were used (exposure time and total irradiance were 50.0 ± 0.1 s and 7.29 ± 0.23 mW/cm^2^, respectively) and the influence of the hydrogel matrix physical thickness on the *DE*_B_ and the angular bandwidth was investigated. For this, hydrogel matrices F prepared in wells with thickness in the range of 120 ± 20 to 570 ± 20 μm were immersed in IS1 and IS6 for 3 h. Transmission holograms were stored in the photohydrogels. Figure 7b shows the *DE*_B_ and the FWHM (full width at half-maximum), obtained from the angular selectivity curves, as a function of the well thickness. A pronounced increase in *DE*_B_ is observed as the well thickness grows from 120 ± 20 to 340 ± 20 μm when both IS1 and IS6 are used in the incubation stage. The maximum *DE*_B_ reached were 36% ± 3% and 42% ± 6% for IS1 and IS6, respectively, corresponding to a well thickness of 460 ± 20 μm. From this thickness, a slight decrease in *DE*_B_ was measured. Regarding the angular selectivity, measured through the FHWM, two different behaviors can be observed. A more pronounced decrease in the FHWM values occurs for well thickness between 120 and 230 ± 20 μm compared to that measured for thickness in the ranges between 340 and 570 ± 20 μm. The highest angular selectivity, i.e., the lower FWHM, was achieved for the higher thicknesses tested. This finding agrees with Kogelnik’s theory.^47^ The angular selectivity is an important parameter, depending on the application for which the photomaterial is designed. For example, in sensing applications, in which the holographic response depends on the concentration of the analyte present in the medium, a high angular selectivity is desired to measure Bragg angle variations more precisely.

### 3.4 Dependence of the Intensity and Exposure Time on the Diffraction Efficiency

**Figure 8:**
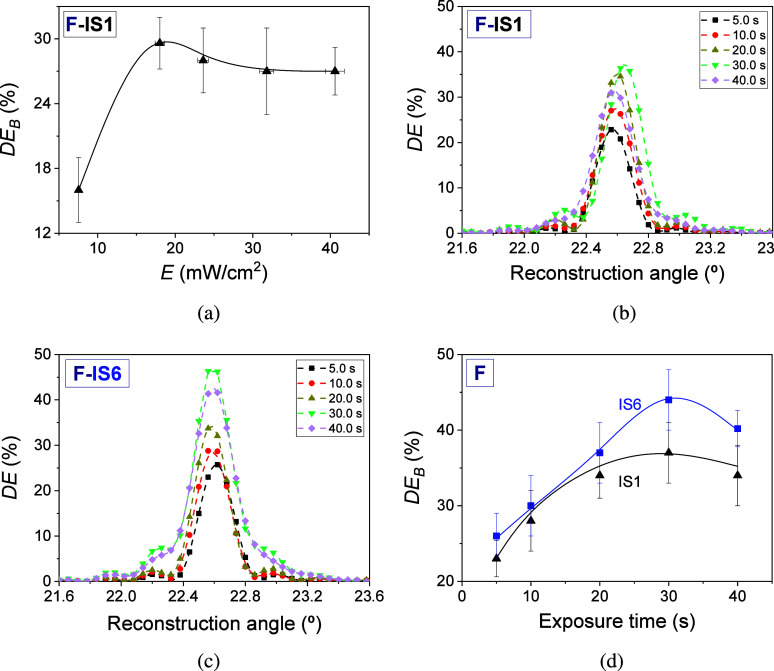
(a) *DE*_B_ as a function of the total irradiance when the exposure time was fixed at 15.0 ± 0.1 s, and IS1 was used in the incubation stage. *DE* as a function of the reconstruction angle for F-IS1 (b) and F-IS6 (c) when exposure times of 5.0 (black box solids), 10.0 (red circle solids), 20.0 (yellow triangle up solids), 30.0 (green triangle down solids), and 40.0 s (violet tilted square solids) were employed. (d) *DE*_B_ as a function of the exposure time when total irradiance was fixed at 18.0 ± 0.6 mW/cm^2^ and IS6 (blue squares) and IS1 (black triangle) were used. Hydrogel matrices F prepared in wells with a thickness of 340 ± 20 μm were employed for preparing the photohydrogels. The solid and dashed lines are guides to the eye.

Figure 7b shows that 460 ± 20 μm is the well thickness at which the best response is obtained in terms of *DE*_B_. However, a slight increase in this thickness causes a decrease in the *DE*_B_. Therefore, and taking into account the calculated errors, a well thickness of 340 ± 20 μm was selected to continue with the study. The next step was to investigate the influence of total irradiance (*E*) and exposure time on *DE*_B_. For this, F matrices prepared in well with a thickness of 340 ± 20 μm were incubated in IS1. First, the resulting photohydrogels were exposed with an exposure time of 15.0 ± 0.1 s while the exposure intensity was varied. The results obtained are listed in Figure 8a. This study used IS1 in the incubation stage. Taking into account the results obtained in the preceding sections 3.13.1–3.3 and the subsequent measurements of the *DE*_B_ values as a function of exposure time, the use of a single incubation solution for optimization of exposure intensity was sufficient. From Figure 8a, it can be seen how the *DE*_B_ values increase with *E* until reaching their maximum value of 29.6 ± 2.4% at an exposure intensity of 18.0 ± 0.6 mW/cm^2^. From this value and when the total irradiance was increased, a slight decrease in *DE*_B_ was measured to remain practically constant up to the maximum total irradiance used. Figure 8b,c shows the angular responses when IS1 and IS6 were used in the incubation stage. From these measurements, *DE*_B_ values were obtained. As expected, the behavior of *DE*_B_ as a function of exposure time is similar for IS1 and IS6 (Figure 8d). The *DE*_B_ values increase until reaching maximum values of 37% ± 4% and 44% ± 4% for IS1 and IS6, respectively, when the exposure time was 30.0 ± 0.1 s. From this value, *DE*_B_ begins to decrease.

To explain this trend, several considerations must be made. As can be seen in Figure 8b,c, the overmodulation regime has not been reached. According to Kogelnik’s theory,^47^ overmodulation phenomena take place when high values of *DE*_B_ (90–100%) are achieved and subsequently a decrease in diffraction efficiency takes place. However, the highest value achieved in our material was 44% ± 4%. The decrease in *DE*_B_ observed in Figure 8d could be explained by considering different processes that take place during the hologram formation stage. All diffusion-based models of hologram formation mechanisms proposed in the literature consider the interplay between monomer polymerization and monomer diffusion.^48−52^ If a cross-linker is included in the composition of the material, as in the case of our photohydrogels, its diffusion must also be taken into account. When the holographic material is exposed to an interference pattern of light, a concentration gradient of the monomer, cross-linker, and polymer is created inside the material. Diffusion of monomer and cross-linker molecules from the unexposed areas to the exposed areas occurs. The variation of the concentration of monomer and cross-linker with exposure time is a consequence of the two mentioned mechanisms: monomer diffusion and monomer polymerization. Since the refractive indices of the formed polymer, monomer, and cross-linker are different, their concentrations play an important role in the hologram formation mechanism. The decrease in *DE*_B_ has been observed in volume transmission diffraction gratings stored in acrylamide PVA based photopolymer.^53^ In this study, the development of a model based on the first harmonic was proposed to explain the formation mechanism of the holograms. This model predicts that when the diffusion times of the monomers are greater than the polymerization rate, a decrease in refractive index modulation occurs after reaching a maximum when this parameter is measured as a function of the exposure time. Most of the monomer is consumed before it can reach the exposed areas, and the modulation of the polymer concentration decreases. On the other hand, it is also necessary to consider the decrease in the modulation of the refractive index produced by the diffusion of short polymer chains created during the illumination of the material from exposed zones to unexposed.^54^ The nonlocal response diffusion model^55^ contemplates that the growth of photopolymer chains away from their point of initiation implies an extension of the photomaterial. All of these processes imply a decrease in the modulation of the refractive index. By fitting the angular scans shown in Figure 8b,c with Kogelnik equations, refractive index modulation values can be obtained. However, the use of these equations would not be correct for our material. As can be seen in Figure 8b,c, a deformation of the side lobes occurs due to the bending and attenuation of the interference fringes. Both processes cause a decrease in the refractive index modulation, which could explain the behavior observed in Figure 8d. Kogelnik’s theory does not predict this behavior and it is necessary to consider more factors to explain the behavior of holograms stored in photohydrogels. Thus, in the following section, a simple model is developed with which the angular scans have been fitted, obtaining the optical parameters of the stored holograms.

### 3.5 Model Based on the Theories of Kogelnik, Kubota, and Uchida to Understand the Role of Bending and Attenuation in Depth in the Behavior of Holograms

The bending of the interference fringes in holograms was theoretically studied and investigated in holograms stored in photographic emulsion by Kubota.^56^ Taking into account the previous works and the study of the grating attenuated holograms by Uchida,^57^ a modification of the Kogelnik’s coupled wave theory^47^ was used for the theoretical analysis of the experimental results. The bending affects the diffraction efficiency, wavelength of the maximum diffraction efficiency, and the angular sensitivity. These parameters are used as signal transducers in holographic sensors in the transmission mode. Theoretically, a decrease in the *DE*_B_ and a shift in the Bragg angle are produced when the bending takes place. Therefore, the optical behavior of the holograms must be well addressed. Taking into account the theories of Uchida and Kubota, the refractive index can be obtained through of next equation:
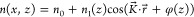
1where *n*_0_ is the average refractive index, *n*_1_ represents the modulation of the refractive index,  is the grating vector (when the bending is zero),  is the position vector, and *z* is dimension in the direction of the thickness of the hologram. The function φ(*z*) denotes the bending of the interferential planes. φ(*z*) can be defined by a polynomial function of different orders. In our model, φ(*z*) has been defined as
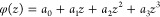
2

The *a*_i_ coefficients are constants that must be entered. On the other hand, the following equation has been proposed to describe the term *n*_1_(*z*):
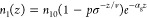
3where *n*_10_ represents the refractive index modulation at *z* = 0; *α*_g_ is the attenuation coefficient of the medium in the recording stage; and *p*, σ, and *v* are constants that must be entered. Once the functions describing the bending and attenuation of the refractive index modulation have been defined, Kogelnik’s coupled wave theory can be used to obtain diffraction efficiency values. In the Kogelnik theory, only two waves propagate in the reconstruction stage of the hologram, the diffracted wave *S*(*z*) and the transmitted wave *R*(*z*). Both waves exchange energy when they propagate through the holographic medium. Following the treatment by Kogelnik, the coupled wave equations are obtained:
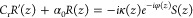

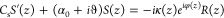
4where *C*_r_ and *C*_s_ are the slant factors, α_0_ is the average absorption coefficient, ϑ is the dephasing measure, and *k*(*z*) is the coupling constant. *C*_r_, *C*_s_*ϑ,* and *k*(*z*) are calculated as
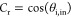
5
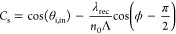
6
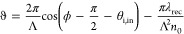
7
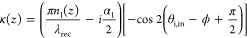
8where λ_rec_ is the reconstruction wavelength (in the air), α_1_ represents the modulation of the absorption coefficient, *θ*_i,in_ is the angle of incidence measured inside the material, Λ is the period of the hologram, and ϕ is the inclination of the interference fringes with respect to the normal to the photohydrogel layer. Considering the boundary conditions for transmission holograms, *R*(0) = 1 and *S*(0) = 0, the system of differential equations can be solved numerically and *DE* is obtained as
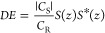
9where S*(*z*) is the complex conjugate of the diffracted wave *S*(*z*).

**Figure 9:**
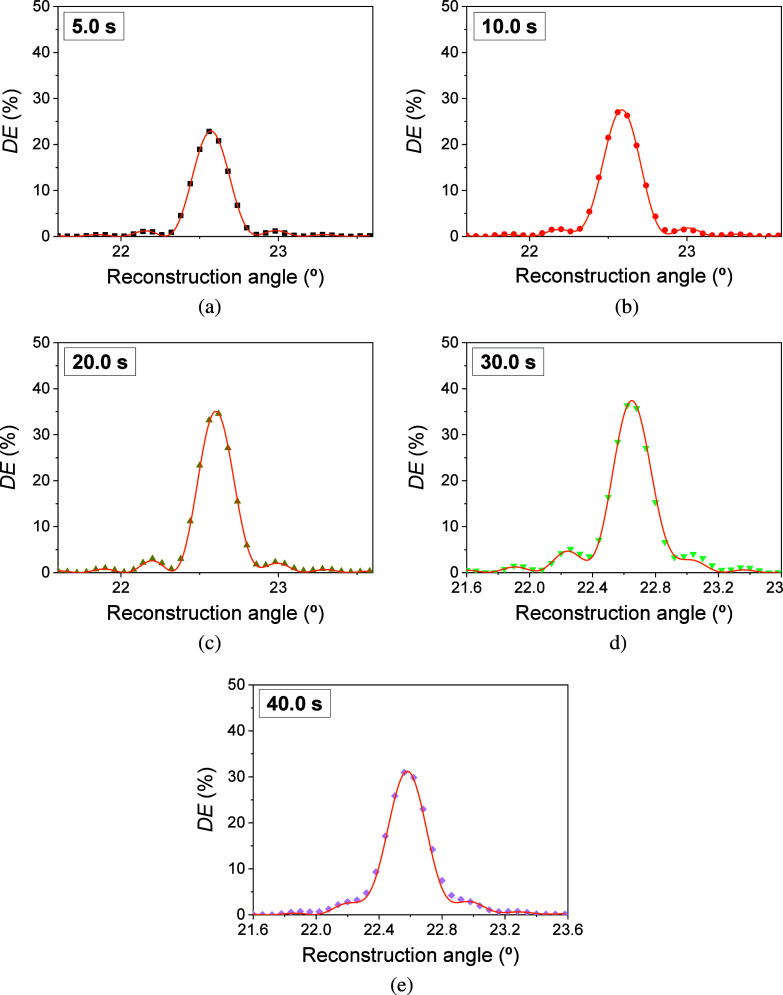
*DE* as a function of the reconstruction angle for F-IS1 when exposure times of 5.0 (a), 10.0 (b), 20.0 (c), 30.0 (d), and 40.0 s (e) were employed. Symbols represented the experimental data and solid lines represented the theoretical fit through the proposed model. The well thickness and total irradiance were 340 ± 20 μm and 18.5 ± 0.6 mW/cm^2^, respectively.

**Figure 10:**
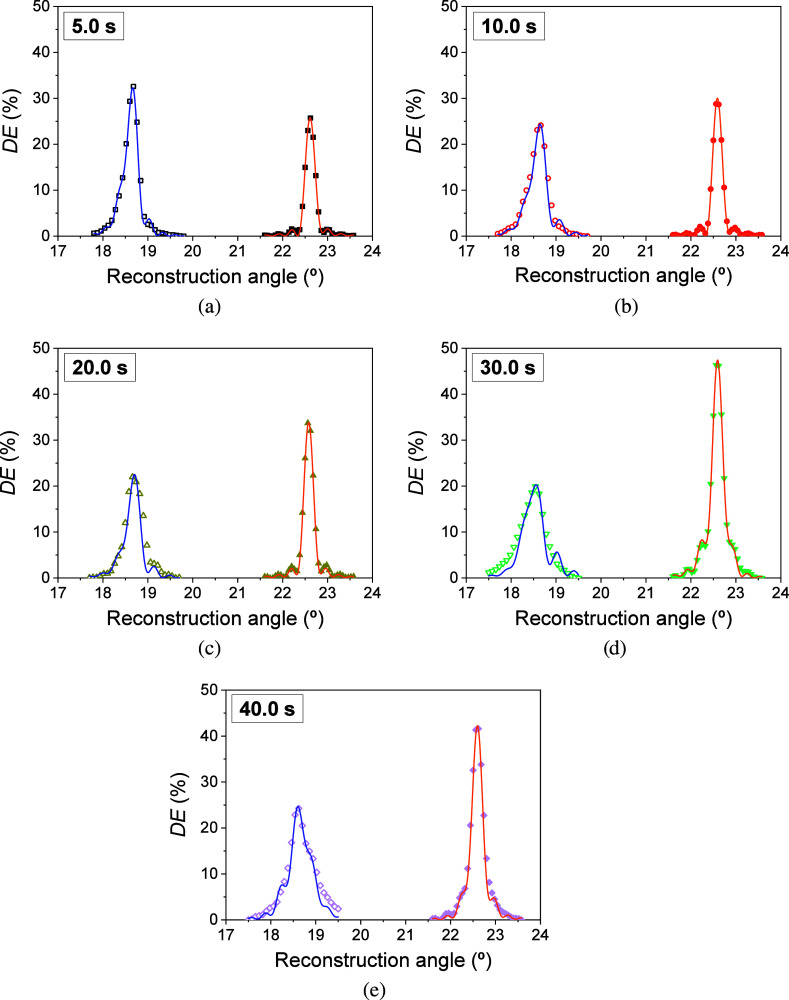
*DE* as a function of the reconstruction angle for F-IS6 when exposure times of 5.0 (a), 10.0 (b), 20.0 (c), 30.0 (d), and 40.0 s (e) were employed. Filled and empty symbols represented the experimental data obtained immediately after exposure (W0) and after washing with PBST (WPBST), respectively. Solid lines represented the theoretical fit through the proposed model. The well thickness and the total irradiance were 340 ± 20 μm and 18.5 ± 0.6 mW/cm^2^, respectively.

Using the proposed model, the angular scans shown in Figure 8c,d were fitted. Figure 9 shows the theoretical fits when IS1 is used in the incubation stage. Since in section 3.6 a comparison is carried out between the diffraction efficiency obtained immediately after the incubation stage and after the washing stage in F-IS6 holograms, the theoretical fits when IS6 is used are shown in Figure 10. As can be seen in both cases, the model presents good agreement with the experimental data, which demonstrates the validity of the equations used to describe both processes, bending and attenuation. Asymmetries in the side lobes due to the combination of bending and gradient attenuation in depth are visible from exposure times of 20.0 s. From this time onward, the lateral lobes begin to increase and merge with the central lobe. This behavior is more visible for an exposure time of 40 s.

It should be noted that the theoretical fits carried out imply considerable difficulty given the number of parameters necessary to introduce into the model. To solve this complexity, different simulations were carried out to understand the operation of the equations for φ(*z*) and *n*_1_(*z*). A summary of the optical parameters obtained through the theoretical fit of the experimental data for holograms F-IS1 and F-IS6 is presented in Tables 3 and 4, respectively. The *n*_0_ values obtained are between 1.423 and 1.430. These values agree with those measured experimentally (∼1.43 at 589 nm). The different AAm/MBA molar ratio in both incubation solutions affects α_0_. In general, α_0_ is smaller for IS1 than for IS6. Anomalous α_0_ values were obtained for exposure times of 40 s in F-IS6. In Figures 9e and 10e, it can be seen that the fusion of the lateral lobes with the central lobe makes the fits made difficult. In future work, research will be carried out to clarify this particular result. The inclination of the interference fringes values with respect to the normal to the photohydrogel layer was maintained at 180° in all theoretical fits. The simulations indicate that the changes made in this parameter do not explain the angular scans obtained. Optical thickness (*d*) increases as the exposure time increases for both incubation solutions. If the values of *d* are compared between IS1 and IS6 for the same exposure times, it is possible to observe how *d* is greater for IS6. This same trend is contemplated for the *n*_10_ values. This parameter represents the refractive index modulation at the surface of the photohydrogels. Therefore, it would not be correct to justify the experimental results obtained only by the product *n*_10_*d*, as would be carried out when only the Kogelnik equation is used. The coefficients *a*_i_ and the constants *p*, σ, and *v* were properly examined to achieve the best fits. For simplicity, these values are not shown in the Tables 3 and 4. For exposure times of 5 and 10 s, the values of *a*_2_ and *a*_3_ were very small. In general, it has been observed that *p*, σ, and *v* acquire larger values as the exposure time increases. This causes the diffraction efficiency to be affected and the decrease of the *DE*_B_ values. In many of the holograms stored with small exposure times, the value of constant *p* introduced in the model was 0. According to eq 3, the refractive index modulation would be governed only by the decaying exponential and the values of α_g_. The different values that this parameter takes depending on the incubation solution used can be clearly observed. Taking into account that as the exposure time increases, so does the bending, and with the results obtained from the theoretical fits, we can propose that the behavior of the holograms stored in our hydrogels is very sensitive to bending. This process may explain the decrease in *DE*_B_ observed in Figure 8d. Furthermore, the interplay between monomer diffusion, monomer polymerization, and chain polymer diffusion must also be taken into account, as was commented in the previous section.

**Table 3:** Parameters Obtained from the Theoretical Fit of the Experimental *DE* Values for F-IS1

Exposure time (s)	*n*_0_	α_0_ × 10^–4^ (μm^–1^)	Λ (μm)	*d* (μm)	*n*_10_ × 10^–4^	α_g_ × 10^–3^ (μm^–^1)
5.0	1.428	1.0	0.8245	240	6.05	1.26
10.0	1.427	1.0	0.8240	241	6.70	1.22
20.0	1.430	1.4	0.8235	245	7.00	0.96
30.0	1.428	1.0	0.8218	235	8.70	0.97
40.0	1.430	1.6	0.8240	255	9.40	1.18

**Table 4:** Parameters Obtained from the Theoretical Fit of the Experimental *DE* Values for F-IS6

Exposure time (s)	*n*_0_	α_0×_10^–4^ (μm^–1^)^1^	Λ (μm)	*d* (μm)	*n*_10_ × 10^–4^	α_g_ × 10^–3^ (μm^–1^)
5.0	1.423	2.2	0.8225	249	6.90	2.40
10.0	1.423	2.2	0.8242	258	7.25	2.30
20.0	1.423	2.6	0.8240	260	9.00	3.50
30.0	1.430	2.3	0.8240	270	9.10	2.60
40.0	1.428	1.1	0.8239	257	10.30	2.60

### 3.6 Behavior of the Diffraction Efficiency After the Washing Stage

Once the hydrogel matrix composition and thickness, the incubator solution composition, and the exposure conditions were set up, the washing to remove the unreacted molecules was also studied. Once the hologram is formed, cross-linked polymer chains tend to diffuse toward unexposed areas while unreacted molecules diffuse toward exposed areas, i.e., areas where interference from laser beams is constructive. The generated concentration gradient produces that the diffraction efficiency decreases, and therefore, it must be removed in order to provide temporal stability to the stored holograms. Different techniques such as exposure to ultraviolet light, dehydration of the photopolymer layers, and incoherent light (LED lamp) have been used to carry out this process.^58,59^ However, a method based on a series of continuous washings of the photohydrogel using PBST buffer was shown to be more convenient.^38^ The influence of the washing stage of the photohydrogels postrecording on *DE*_B_ was investigated. Photohydrogels prepared from matrices F (well thickness of 340 ± 20 μm) and incubated with IS6 were exposed with a total irradiance of 18.5 ± 0.6 mW/cm^2^. In order to study the possible different behavior of holograms stored with different values of radiant exposure, first, exposure times from 5.0 ± 0.1 to 40 ± 0.1 s were employed. The washing of the postrecording photohydrogels was carried out with a PBST buffer following the procedure described in section 2.5. *DE* as a function of the reconstruction angle was measured immediately after the exposure stage (W0) and after washing with PBST (WPBST). The size of the holograms increased to 4.9 ± 0.1 mm after the washing stage due to the swelling of the hydrogels when immersed in PBST. The angular scans of transmission gratings for both stages and the theoretical fits using the model presented in the previous section are shown in Figure 10. As can be clearly observed, the bending of the interference fringes is more visible in WPBST. The left lobe is more intense and is more fused to the central lobe, compared to stage W0, for exposure times from 5 to 30 s (Figure 10a–d). This behavior is the opposite when a time of 40 s is used (Figure 10e). In this case, the most intense lobe is the left one. This behavior does not follow a clear trend. In other measurements with the same exposure times, variation has been found in the shape of the angular scans for WPBST. The diffraction efficiency is affected by the bending, which depends on different factors such as the type of solvent used for washing.^38^

A summary of the optical parameters obtained through the theoretical fit for holograms F-IS6 after washing is presented in Table 5. The solvent used in the incubation solutions is DMSO:H_2_O (6:4) while washing is carried out with PBST buffer. This change of medium produces a swelling of the hydrogel, causing an angular shift with respect to the reconstruction angle. The interferential fringes widen and the period of the stored holograms increases. A Bragg angle of 18.6 ± 0.1° was measured in WPBST. The *n*_0_ values are between 1.350 and 1.357. Since after the washing stage, the hydrogels contain water inside, the value of *n*_0_ is closer to the value of the refractive index of the water (1.33). Regarding optical thickness, there is an increase in its value, compared to that of W0, for exposure times of 5 and 40 s. The *d* values are similar if we compare those obtained in W0 with those shown for WPBST. This demonstrates once again the variability that bending adopts in this type of materials capable of having a high swelling value. It should be noted that *n*_10_ for WPBST increases with respect to those obtained in W0 when exposure times of 5 and 10 s are used. This trend reverses after 20 s. A clear increase in α_g_ can be observed when comparing both stages, W0 and WPBST. This phenomenon requires more research, which will be carried out in future works.

**Table 5:** Parameters Obtained From the Theoretical Fit of the Experimental *DE* Values when the Washing Stage is Carried Out in Photohydroges F-IS6

Exposure time (s)	*n*_0_	α_0_ × 10^–4^ (μm^–1^)	Λ (μm)	*d* (μm)	*n*_10_ × 10^–4^	α_g_ × 10^–3^ (μm^–1^)
5.0	1.357	1.0	0.9860	301	6.90	3.59
10.0	1.357	2.0	0.9900	254	8.10	3.38
20.0	1.357	2.0	0.9950	256	7.80	3.55
30.0	1.359	1.0	0.9950	254	8.0	3.09
40.0	1.350	1.0	0.9900	271	8.55	2.55

**Figure 11:**
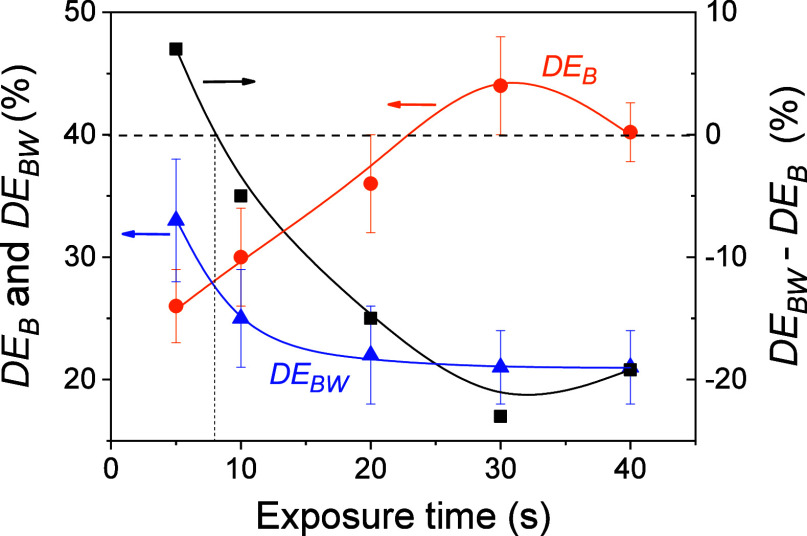
*DE*_B_ and *DE*_BW_ (left axis) and *DE*_BW_ – *DE*_B_ (right axis) as a function of the exposure time for transmission gratings stored in hydrogel matrices F incubated with IS6. The well thickness and the total irradiance were 340 ± 20 μm and 18.5 ± 0.6 mW/cm^2^, respectively. The solid lines are guides to the eye.

The *DE*_B_ behavior in WPBST (*DE*_BW_) was different, depending on the exposure time used. *DE*_B_ increases from 26% ± 3% in W0 to 33% ± 5% in WPBST for an exposure time of 5.0 ± 0.1 s. However, this behavior was reversed when the exposure time is increased to 10.0 ± 0.1 s. In this case, a *DE*_B_ of 30% ± 4% was reached for W0 while it decreased to a value of 25% ± 4% when the washing stage is carried out. Figure 11 shows the *DE*_B_ and *DE*_BW_ values for different exposure times. *DE*_BW_ values decrease rapidly when exposure times from 5.0 ± 0.1 to 20.0 ± 0.1 s were used. From here, *DE*_BW_ decays slightly from 22% ± 4% to 21% ± 3% for exposure times of 20.0 ± 0.1 and 40.0 ± 0.1 s, respectively. The calculation of the quantity *DE*_BW_ – *DE*_B_ allows us to appreciate more clearly the behaviors of the holograms stored in the photohydrogels. This quantity is represented on the left axis in Figure 11. Changes in diffraction efficiency after the washing stage do not occur when the *DE*_BW_ – *DE*_B_ quantity is zero. This occurs for an exposure time of about 8 s. For longer exposure times, *DE*_BW_ – *DE*_B_ was negative and the hologram presents a lower diffraction efficiency in WPBST. The highest difference between *DE*_BW_ and *DE*_B_ was measured for an exposure time of 30.0 ± 0.1 s. The different amounts of bending produced after the washing stage depending on the exposure time can explain this behavior. Furthermore, it must be taken into account that in the exposure stage, the density of the AAm–MBA polymeric network formed in the exposed areas is higher as the exposure time increases, i.e., larger radiant exposure. This results in a greater modulation of the refractive index. Consequently, a higher *DE* value is obtained. During the washing stage, the unreacted components in the unexposed areas are removed. When washing is complete, a further modulation of the refractive index is produced.

## 4 Conclusions

Optimization of the storage of unslanted transmission volume phase holographic gratings in photohydrogels based on AAm and MBA was performed. First, hydrogel matrices with different degrees of cross-linking and the same thickness were synthesized. Images obtained by scanning electron microscopy show a homogeneous nonmicroporous structure for all hydrogels. In the case of the matrices with higher degrees of cross-linking (A and B), pores of nanometric size were observed. The transmittance measurements of the hydrogel layers showed that those matrices with the lowest degree of cross-linking were the ones that presented the highest transparency (F and G). The behavior of the diffraction efficiency as a function of the degree of cross-linking was studied. The highest diffraction efficiencies were obtained for the hydrogel matrix with AAm/MBA molar ratios of 19.9 (E), 22.6 (F), and 26 (G), reaching a maximum of 30% ± 5% for the F matrix. Once the best hydrogel matrices in terms of diffraction efficiency were selected, the AAm/MBA molar ratio in the incubation solutions was optimized. The highest diffraction efficiency, around 35%, was achieved when an incubation solution with an AAm/MBA molar ratio of 4.35 (IS6) was used. The influences of the hydrogel matrix physical thickness, the intensity, and the exposure time on the diffraction efficiency were also investigated and optimized. The results indicated that the highest values of diffraction efficiency, 44% ± 4%, were reached when the well thicknesses were between 340 and 460 μm, and the intensity and exposure time were 18.0 ± 0.6 mW/cm^2^ and 30.0 ± 0.1 s, respectively. Finally, the behavior of the holographic gratings in the washing stage was analyzed. The diffraction efficiencies measured immediately after the exposure and the PBST wash were similar when an exposure time of approximately 8 s was employed. For longer exposure times, the diffraction efficiency after the washing stage presents lower values compared to those obtained during the exposure stage. A simple model was proposed and used to fit the experimental data. This model considered the bending and attenuation of holographic gratings in depth. The results obtained present good agreement with the proposed model, demonstrating that the behavior of holograms stored in photohydrogels is very sensitive to both effects. This study demonstrates that optimization of hologram storage in photohydrogels is key for the further development of photonic devices such as holographic sensors.
